# Draft genome sequence of *Halomonas lutea* strain YIM 91125^T^ (DSM 23508^T^) isolated from the alkaline Lake Ebinur in Northwest China

**DOI:** 10.1186/1944-3277-10-1

**Published:** 2015-01-20

**Authors:** Xiao-Yang Gao, Xiao-Yang Zhi, Hong-Wei Li, Yu Zhou, Alla Lapidus, James Han, Matthew Haynes, Elizabeth Lobos, Marcel Huntemann, Amrita Pati, Natalia N Ivanova, Konstantinos Mavromatis, Brian J Tindall, Victor Markowitz, Tanja Woyke, Hans-Peter Klenk, Nikos C Kyrpides, Wen-Jun Li

**Affiliations:** 1 Key Laboratory of Biogeography and Bioresource in Arid Land, Xinjiang Institute of Ecology and Geography, Chinese Academy of Sciences, Urumqi, China; 2 Key Laboratory of Microbial Diversity in Southwest China, Ministry of Education and the Laboratory for Conservation and Utilization of Bio-Resources, Yunnan Institute of Microbiology, Yunnan University, Kunming, China; 3 Theodosius Dobzhansky Center for Genome Bionformatics, St. Petersburg State University, St. Petersburg, Russia; 4 Algorithmic Biology Lab, St. Petersburg Academic University, St. Petersburg, Russia; 5 DOE Joint Genome Institute, Walnut Creek, California, USA; 6 Biological Data Management and Technology Center, Lawrence Berkeley National Laboratory, Berkeley, California, USA; 7 Leibniz-Institute DSMZ - German Collection of Microorganisms and Cell Cultures, Braunschweig, Germany; 8 Department of Biological Sciences, King Abdulaziz University, Jeddah, Saudi Arabia; 9 The First Hospital of Qujing City, Qujing Affiliated Hospital of Kunming Medical University, Qujing, China; 10 State Key Laboratory Breeding Base for Zhejiang Sustainable Plant Pest Control, Institute of Quality and Standard for Agro-products, Zhejiang Academy of Agricultural Sciences, Hangzhou, Zhejiang, China; 11 University of Chinese Academy of Sciences, Beijing, China; 12 School of Biology, Newcastle University, Newcastle upon Tyne, UK

**Keywords:** *Halomonas lutea*, Aerobic, Gram-negative, Chemoorganotrophic, Moderately halophilic, Lake Ebinur

## Abstract

Species of the genus *Halomonas* are halophilic and their flexible adaption to changes of salinity and temperature brings considerable potential biotechnology applications, such as degradation of organic pollutants and enzyme production. The type strain *Halomonas lutea* YIM 91125^T^ was isolated from a hypersaline lake in China. The genome of strain YIM 91125^T^ becomes the twelfth species sequenced in *Halomonas*, and the thirteenth species sequenced in *Halomonadaceae.* We described the features of *H. lutea* YIM 91125^T^, together with the high quality draft genome sequence and annotation of its type strain. The 4,533,090 bp long genome of strain YIM 91125^T^ with its 4,284 protein-coding and 84 RNA genes is a part of Genomic Encyclopedia of Type Strains, Phase I: the one thousand microbial genomes (KMG-I) project. From the viewpoint of comparative genomics, *H. lutea* has a larger genome size and more specific genes, which indicated acquisition of function bringing better adaption to its environment. DDH analysis demonstrated that *H. lutea* is a distinctive species, and halophilic features and nitrogen metabolism related genes were discovered in its genome.

## Introduction

Strain YIM 91125^T^ (= DSM 23508^T^ = KCTC 12847^T^ = CCTCC AB 206093^T^) is the type strain of *Halomonas lutea*[1]. Currently, there are 83 validly named species in the genus *Halomonas* on the basis of most recent released from LPSN [2] and EzTaxon-e [3]. *Halomonadaceae* comprises the largest number of halophilic and halotolerant bacteria described to date, and *Halomonas* is the largest genus in this family. However, most of the taxa in *Halomonadaceae* have been reclassified in the past due to their heterogeneous features [4-7]. In *Halomonas*, a small group of species has been formally re-located to *Chromohalobacter*, *Cobetia* and *Kushneria* by further taxonomic studies. Members of the genus *Halomonas* were usually isolated from saline environments [8-12]. Strain YIM 91125^T^ was originally isolated from soil sample of Ebinur Lake, which has been a long-term target for the studies of element cycling and microbial biota under extremely high-saline conditions in Xinjiang, Northwest China. As a type strain, it’s the original isolate used in species description, which exhibits the relevant phenotypic and genotypic properties cited in the original published taxonomic circumscriptions [13]. This organism grows well across a wide range of salinity and temperature and also participates in nitrogen reduction. In this context, strain YIM 91125^T^ has been sequenced as a halophilic representative, and becomes a part of Genomic Encylopedia of Type Strains, Phase I: the one thousand microbial genomes project.

Here, we present a summary classification and a set of features for *H. lutea* strain YIM 91125^T^, together with the description of the genomic sequencing and annotation, and provide brief findings of its genome sequence as compared to genomes of other *Halomonas* species. The genomic data will provide insights into its new biotechnological applications, such as sewage treatment. The comprehensive genomes of this genus will facilitate our understanding of the ecological roles that *Halomonas* species play in those hypersaline habitats and their relationships with other halophilic and nonhalophilic microorganisms.

### Classification and features

**Figure 1:**
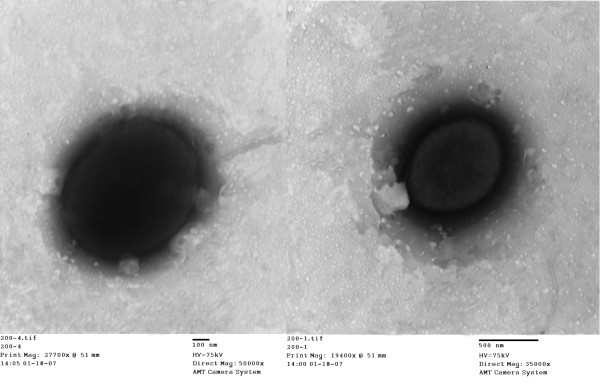
**Transmission electron micrograph of *H. lutea *** **YIM 91125**^**T**^**.**

*H. lutea* YIM 91125^T^ is a Gram-negative-staining, motile, aerobic and moderately halophilic bacterium, which can reduce nitrate (Table 1). Cells of the strain are short rods, 0.4 to 0.7 μm in diameter and 0.6 to 1.0 μm in length (Figure 1). They are motile by means of single polar flagellum and their colonies are orange, flat, opaque and mucoid with slightly irregular edges (Figure 1). The predominant respiratory quinone found in *H. lutea* YIM 91125^T^ is Q-9, similar to other members of the genus *Halomonas*. The predominant fatty acids are C_18:1_*ω*7*c* (25.1%), C_16:0_ (17.0%), C_19:0_ cyclo *ω*8*c* (13.6%), C_12:0_ 3-OH (10.7%), C_12:0_ (7.9%), C_10:0_ (6.0%) and C_17:0_ cyclo (4.6%) [1]. The profile of major fatty acids in strain YIM 91125^T^ is also similar to other members of the genus *Halomonas*[14-17].

**Table 1:** **Classification and general features of *H. lutea *** **YIM 91125**^**T**^[18]

**MIGS ID**	**Property**	**Term**	**Evidence code**^**a**^
	Classification	Domain *Bacteria*	TAS [19]
		Phylum *Proteobacteria*	TAS [20]
		Class *Gammaproteobacteria*	TAS [21,22]
		Order *Oceanospirillales*	TAS [21,23]
		Family *Halomonadaceae*	TAS [4]
		Genus *Halomonas*	TAS [24]
		Species *Halomonas lutea*	TAS [1]
		Type strain YIM 91125^T^	TAS [1]
	Gram stain	negative	TAS [1]
	Cell shape	short rods	TAS [1]
	Motility	motile	TAS [1]
	Sporulation	non-sporulating	TAS [1]
	Temperature range	4-45°C	TAS [1]
	Optimum temperature	37°C	TAS [1]
	pH range; Optimum	5.0-9.0	TAS [1]
	Carbon source	mono- and polysaccarides	TAS [1]
MIGS-6	Habitat	aquatic, fresh water, lake, salinewater	TAS [1]
MIGS-6.3	Salinity	1-20% NaCl (w/v)	TAS [1]
MIGS-22	Oxygen requirement	aerobe	TAS [1]
MIGS-15	Biotic relationship	free living	TAS [1]
MIGS-14	Pathogenicity	none	NAS
MIGS-4	Geographic location	Ebinur Lake (China)	TAS [1]
MIGS-5	Sample collection	2008 or before	NAS
MIGS-4.1	Latitude	45.05	TAS [1]
MIGS-4.2	Longitude	82.977	TAS [1]
MIGS-4.4	Altitude	not reported	

^a^Evidence codes – TAS: Traceable Author Statement (i.e., a direct report exists in the literature); NAS: Non-traceable Author Statement (i.e., not directly observed for the living, isolated sample, but based on a generally accepted property for the species, or anecdotal evidence). These evidence codes are from the Gene Ontology project [25].

16S rRNA gene sequence of strain YIM 91125^T^ was compared with the newly released database from the Greengenes [26], using NCBI BLAST [27,28] under default settings (e.g., considering only HSPs from the best 250 hits) and the relative frequencies of taxa were determined, weighted by BLAST scores. The most frequently occurring genera were *Halomonas* (71.4%), *Chromohalobacter* (17.8%), *Bacillus* (3.6%), *Haererehalobacter* (3.6%) and *Modicisalibacter* (3.6%) (228 hits in total). Regarding 186 hits to sequences from members of the genus *Halomonas*, the average identity within HSPs was 95.5%, whereas the average coverage by HSPs was 98.3%. Among all other species, the one yielding the highest score was *Halomonas xinjiangensis*, which corresponded to identity of 99.9% and HSP coverage of 98.0%. (Note that the Greengenes database uses the INSDC (=EMBL/NCBI/DDBJ) annotation, which is not an authoritative source for nomenclature or classification.) The highest scoring environmental sequences were EF157249 and EF157230 (Greengenes short name ‘tar pits clone 101–11 k’ and ‘tar pits clone 101–120 k’), which showed identity of 96.3% and an HSP coverage of 99.6%. The most frequently occurring keywords within the labels of all environmental samples which yielded hits were soil like ‘soil’, ‘seafloor’, ‘drilling deep-earth’; water like ‘groundwater’, ‘aquatic’, ‘lake’, ‘marine’; oil and plant. Environmental samples yielded hits of a higher score than the highest scoring species were not found.

**Figure 2:**
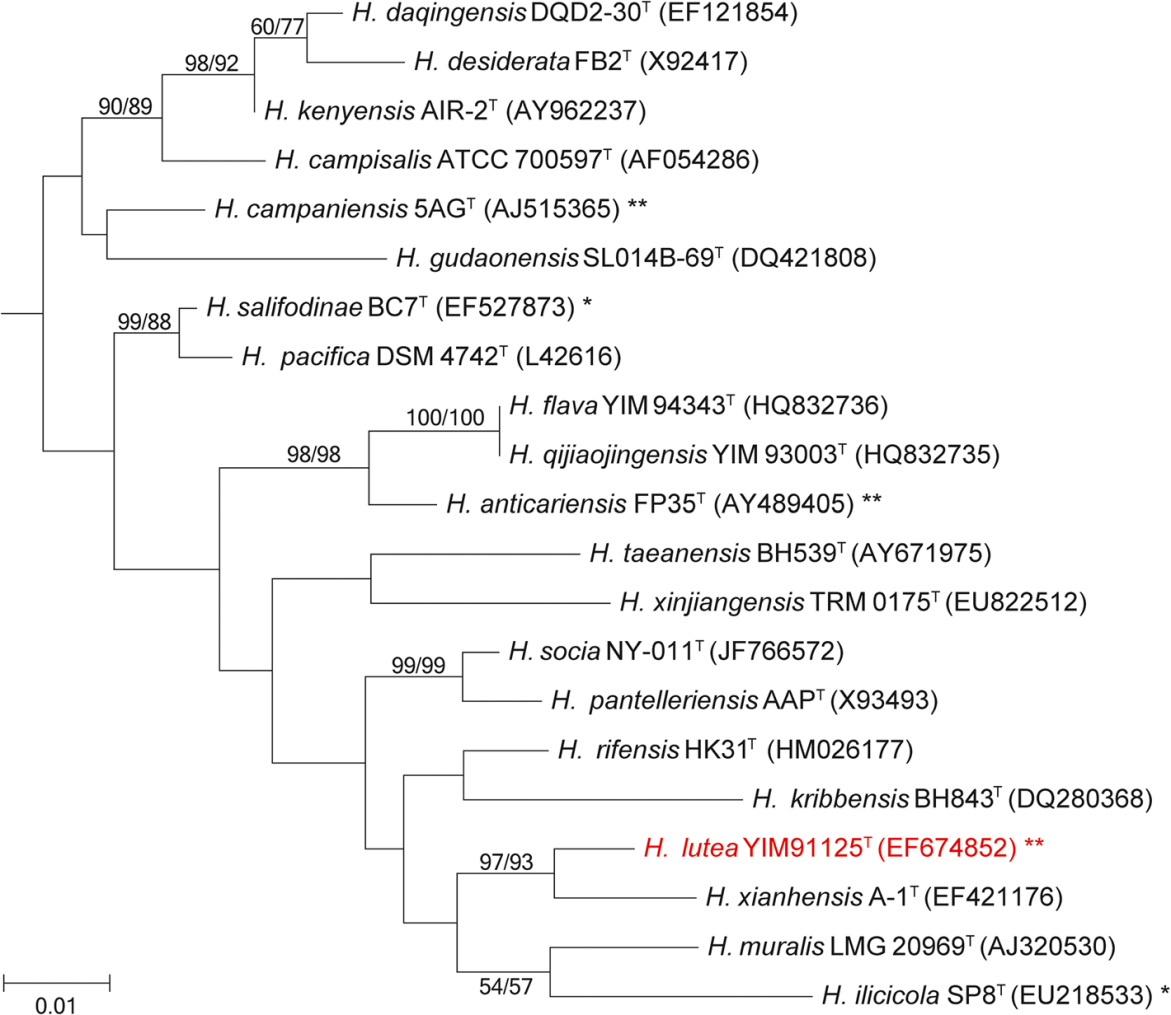
**Phylogenetic tree highlighting the position of *H. lutea *relative to the type strains of the other species within *Halomonas*.** According to the most recent release of the EzTaxon-e database, all the 16S rRNA gene sequences of the type strains within genus *Halomonas* were retained. The tree was inferred from 1,383 aligned bases [29] under the neighbor-joining (NJ) [30] and maximum-likelihood (ML) [31] methods with 1,000 randomly selected bootstrap replicates using MEGA version 5.2 [32]. The branches are scaled in terms of the expected number of substitutions per site. Numbers adjacent to the branches are support values from 1,000 NJ bootstrap (left) and from 1,000 ML bootstrap (right) replicates [33] if they are larger than 50%. Lineages with type strain genome sequencing projects registered in Genomes OnLine Database (GOLD) [34] are labeled with one asterisk, and those have available genomic data are labeled with two asterisks. Non-type strain LS21of *H. campaniensis* and *H. elongata* DSM 2581^T^ listed ‘Complete and Published’ are also labeled with two asterisks.

Phylogenetic analyses were carried out with two different algorithms, i.e., neighbor-joining (NJ) and maximum-likelihood (ML). The phylogenetic tree was shown in Figure 2 and Additional file 1: Figure S1, which provides an interesting insight into the nomenclature and classification of members of the genus *Halomonas*, and also indicates the phylogenetic neighborhood of *H. lutea*. The phylogenetic relationships indicate that *H. lutea* YIM 91125^T^ is most closely to *H. xianhensis* A-1^T^ with 99% similarity and the sequence of the sole 16S rRNA gene in the genome differs by 10 nucleotides from the previously published 16S rRNA sequence (EF674852).

## Genome sequencing and annotation

### Genome project history

This organism was selected for sequencing on the basis of its phylogenetic position and biological application importance [35,36], and for a better understand the mechanism of its halophilic adaptation. Sequencing of *H. lutea* YIM 91125^T^ is part of Genomic Encyclopedia of Type Strains, Phase I: the one thousand microbial genomes (KMG-I) project [37], a follow-up of the GEBA project [38], which aims for increasing the sequencing coverage of key reference microbial genomes. The genome project is deposited in the Genomes OnLine Database (GOLD), and the high quality draft genome sequence is deposited in GenBank. Sequencing, finishing and annotation were performed by the DOE JGI using state of the art sequencing technology [39]. A summary of the project information is shown in Table 2. It presents the project information and in compliance with MIGS version 2.0 compliance [18].

**Table 2:** Project information

**MIGS ID**	**Property**	**Term**
MIGS-31	Finishing quality	Improved-High-Quality Draft
MIGS-28	Libraries used	Illumina standard shotgun library
MIGS-29	Sequencing platforms	Illumina HiSeq 2000
MIGS-31.2	Fold coverage	119 ×
MIGS-30	Assemblers	Velvet v. 1.1.04; ALLPATHS v. r41043
MIGS-32	Gene calling method	Prodigal 1.4
	Locus Tag	NZ_ARKK01000000
	Genbank ID	ARKK00000000
	Genbank Date of Release	April 23, 2013
	GOLD ID	Gi11553
	BIOPROJECT	PRJNA199405
	Project relevance	Genomic Encyclopedia of Type Strains, Phase I: the one thousand microbial genomes (KMG-I) project
MIGS-13	Source Material Identifier	*Halomonas lutea* DSM 23508

### Growth conditions and DNA isolation

*H. lutea* strain YIM 91125^T^ (DSM 23508^T^), was grown in DSMZ medium 514b (Medium 514 plus additional salt) at 37°C [40]. DNA was isolated from 0.5-1.0 g of cell pasted using Jetflex Genomic DNA Purification Kit (Qiagen, Hilden, Germany), following the standard protocol as recommended by the manufacturer, but with an additional incubation (60 min, 37°C) with 50 μl proteinase K and finally adding 200 μl protein precipitation buffer (PPT). DNA is available through the DNA Bank Network [41].

### Genome sequencing and assembly

The draft genome of strain YIM 91125^T^ was generated at JGI using Illumina technology [42]. An Illumina standard shotgun library was constructed and sequenced using the Illumina HiSeq 2000 platform which generated 9,251,032 reads totaling 1,387.7 Mb. All general aspects of library construction and sequencing performed at the JGI. All raw Illumina sequence data was passed through DUK, a filtering program developed at JGI, which removes known Illumina sequencing and library preparation artifacts. The following steps were then performed for assembly: (1) filtered Illumina reads were assembled using Velvet version 1.1.04 [43]; (2) 1–3 Kb simulated paired end reads were created from Velvet contigs using Wgsim [44]; (3) Illumina reads were assembled with simulated read pairs using Allpaths-LG [45]. The final draft assembly contained 49 contigs in 42 scaffolds. The total size of the genome is 4.5 Mbp and the final assembly is based on 538.9 Mbp of Illumina data, which provides an average 119.0 × coverage of the genome.

### Genome annotation

Genes were identified using Prodigal [46] as part of the DOE JGI genome annotation pipeline [47], following by a round of manual curation using the JGI GenePRIMP pipeline [48]. The predicted CDSs were translated and used to search the NCBI non-redundant database, UniProt, TIGR-Fam, Pfam, PRIAM, KEGG, COG, and InterPro database. These data sources were combined to assert a product description for each predicted protein. Additional gene prediction analysis and functional annotation were performed within the Integrated Microbial Genomes-Expert Review (IMG-ER) platform [49].

### Genome properties

**Figure 3:**
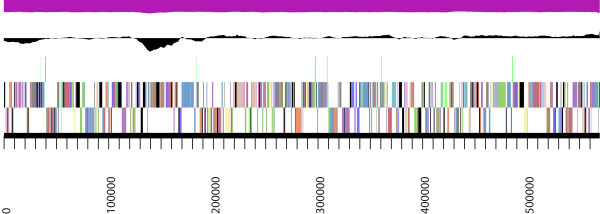
**Graphical map of the largest scaffold in *Halomonas lutea *YIM 91125**^**T**^**.** From bottom to the top: Genes on forward strand (colored by COG categories), Genes on reverse strand (colored by COG catergories), RNA genes (tRNA green, rRNA red, other RNAs black), GC content, GC skew (purpele/olive).

The assembly of the draft genome sequence consists of 42 scaffolds (Figure 3) amounting to 4,533,090 bp, and G+C content is 59.1%. The majority of the protein-coding genes (83.0%) were assigned a putative function while the remaining ones were annotated as hypothetical proteins. 3,325 protein coding genes belong to 422 paralogous families in this genome. The properties and the statistics of the genome are summarized in Table 3. The distribution of genes into COGs functional categories is presented in Table 4.

**Table 3:** Genome statistics

**Attribute**	**Value**
Genome size (bp)	4,533,090
DNA coding (bp)	3.982.279
DNA G + C (bp)	2.676.712
DNA scaffolds	42
Total genes	4,368
Protein-coding genes	4,284
RNA genes	84
Pseudo genes	51
Genes in internal clusters	3,325
Genes with function prediction	3,625
Genes assigned to COGs	3,497
Genes with Pfam domains	3,674
Genes with signal peptides	326
Genes with transmembrane helices	1,033
CRISPR repeats	1

**Table 4:** Number of genes associated with general COG functional categories

**Code**	**Value**	**% age**	**Description**
J	183	4.66	Translation, ribosomal structure and biogenesis
A	1	0.03	RNA processing and modification
K	278	7.08	Transcription
L	168	4.28	Replication, recombination and repair
B	6	0.15	Chromatin structure and dynamics
D	37	0.94	Cell cycle control, Cell division, chromosome partitioning
V	36	0.92	Defense mechanisms
T	208	5.30	Signal transduction mechanisms
M	210	5.35	Cell wall/membrane biogenesis
N	92	2.34	Cell motility
U	80	2.04	Intracellular trafficking and secretion
O	158	4.02	Posttranslational modification, protein turnover, chaperones
C	291	7.41	Energy production and conversion
G	273	6.95	Carbohydrate transport and metabolism
E	352	8.96	Amino acid transport and metabolism
F	85	2.16	Nucleotide transport and metabolism
H	186	4.74	Coenzyme transport and metabolism
I	133	3.39	Lipid transport and metabolism
P	218	5.55	Inorganic ion transport and metabolism
Q	126	3.21	Secondary metabolites biosynthesis, transport and catabolism
R	467	11.89	General function prediction only
S	339	8.63	Function unknown
-	871	19.94	Not in COGs

The total is based on the total number of protein-coding genes in the annotated genome.

### Insights from the genome sequence

**Figure 4:**
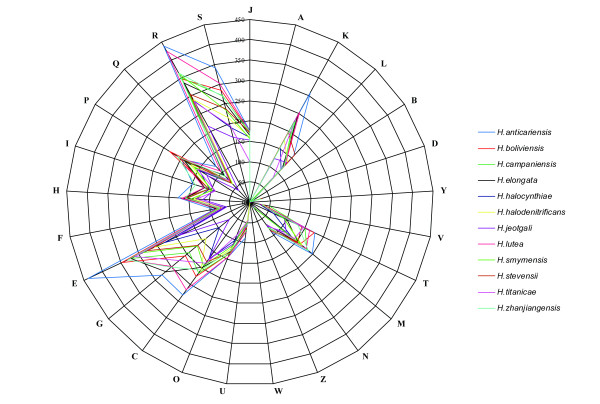
**Distribution of functional classes of predicted genes in *Halomonas *** **species chromosomes according to the clusters of orthologous groups of proteins.**

The genomic sequences of twelve *Halomonas* species are available, including *H. lutea* YIM 91125^T^. Genome properties of those *Halomonas* species are shown in Table 5, but only *H. elongate* and *H. campaniensis* have complete genome sequences. These *Halomonas* genome sequences exhibit dramatic interspecies variations in size, ranging from 5.34 Mb (*H. titanicae*) to 2.85 Mb (*H. jeotgali*); and the size of *H. lutea* is larger than the average size, suggesting acquisition of functions may allow better adaption to its environment, e.g., genes coding for tripartite ATP-independent periplasmic (TRAP) transporters for substrate uptake or nitrate degradation [50]. Also, GC contents of those species vary from 52.65% (*H. campaniensis*) to 67.86% (*H. smyrnensis*), and of *H. lutea* (59.05%) is around the average GC content, close to *H. anticariensis* (58.54%). In addition, the distribution of genes into COG categories was not entirely similar in all twelve compared genomes (Figure 4). And *H. lutea* has more specific genes, since proteins with COG only account for 71.18% which is lower than other members. Compared with other *Halomonas* species, the proportions of genes with signal peptide and transmembrane helices of *H. lutea* are respectively 7.46% and 23.65%, close to the corresponding averages. The abundance of transmembrane helices related genes indicates the important role in metabolism process of *Halomonas*.

**Table 5:** **Comparison of genome features of *Halomonas *** **species**

**Species**	**Genome size (Mb)**	**GC content (%)**	**Gene count**
*H. anticariensis* FP35^T^	5.07	58.54	4817
*H. boliviensis* LC1^T^	4.14	54.68	3915
*H. campaniensis* LS21	4.07	52.65	3665
*H. elongata* DSM 2581^T^	4.06	63.61	3556
*H. halocynthiae* DSM 14573^T^	2.88	53.80	2773
*H. halodenitrificans* DSM 735^T^	3.47	63.95	3256
*H. jeotgali* Hwa^T^	2.85	62.92	2636
*H. lutea* YIM 91125^T^	4.53	59.05	4368
*H. smyrnensis* AAD6^T^	3.56	67.86	3326
*H. stevensiss* S18214^T^	3.69	60.25	3523
*H. titanicae* BH1^T^	5.34	54.58	2908
*H. zhanjiangensis* DSM 21076^T^	4.06	54.48	3739

DNA-DNA hybridization is considered as a gold-standard of distinguishing species [51]. Digital DDH similarities between genome of *H. lutea* and those of other *Halomonas* species were calculated using GGDC web server version 2.0 under recommend setting [52,53]. The probabilities of DDH value > 70% assessed via logistic regression under three formulae indicate that *H. lutea* is different from other species of the genus (Table 6). The inter-genome distances under formula 2 between *H. lutea* and *H. anticariensis*, *H. boliviensi*, *H. campaniensis*, *H. elongata*, *H. halocynthiae*, *H. halodenitrificans*, *H. jeotgali*, *H. smyrnensis*, *H. stevensii*, *H. titanicae* and *H. zhanjiangensis* are about 0.22, the corresponding DDH estimates below the 70% threshold under formula 2 are: 19.5% (± 2.29), 20.2% (± 2.31), 21.1% (± 2.33), 20.1% (± 2.31), 19.2% (± 2.29), 19.4% (± 2.29), 19.9% (± 2.30), 20.3% (± 2.32), 20.4% (± 2.32), 20.5% (± 2.32), 18.9% (± 2.28), respectively. The standard deviations indicate the inherent uncertainty in estimating DDH values from intergenomic distances based on models derived from empirical test data sets. Given that the low degree of DNA-DNA similarity among *Halomonas* species, it appears justified to assume that these strains represent different species. For better understanding of the relationships between *H. lutea* and other *Halomonas* members, availability of more genome sequences of representatives are needed to implement phylogenomic inference.

**Table 6:** **Digital DDH similarities between *H. lutea *** **DSM 23529**^**T**^**and the other *Halomonas s*** **pecies**

**Reference species**	**Formula 1**	**Formula 2**	**Formula 3**
*H. anticariensis*	14.9 ± 3.14	19.5 ± 2.29	15.0 ± 2.67
*H. boliviensis*	13.0 ± 2.99	20.2 ± 2.31	13.4 ± 2.56
*H. campaniensis*	13.0 ± 2.99	21.1 ± 2.33	13.3 ± 2.56
*H. elongata*	15.6 ± 3.19	20.1 ± 2.31	15.6 ± 2.70
*H. halocynthiae*	13.0 ± 2.99	19.2 ± 2.29	13.3 ± 2.56
*H. halodenitrificans*	14.5 ± 3.11	19.4 ± 2.29	14.6 ± 2.65
*H. jeotgali*	13.5 ± 3.03	19.9 ± 2.30	13.8 ± 2.59
*H. smyrnensis*	15.5 ± 3.18	20.3 ± 2.32	15.5 ± 2.70
*H. stevensiss*	13.5 ± 3.04	20.4 ± 2.32	13.8 ± 2.59
*H. titanicae*	13.0 ± 2.99	20.5 ± 2.32	13.3 ± 2.56
*H. zhanjiangensis*	13.2 ± 3.01	18.9 ± 2.28	13.5 ± 2.57

GenBank accession numbers for the reference genomes: *H. anticariensis* (NZ_ASTJ00000000), *H. boliviensi* (NZ_AGQZ00000000), *H. campaniensis* (CP007757), *H. elongata* (NC_014532), *H. halocynthiae* (AUDZ00000000), *H. halodenitrificans* (JHVH00000000), *H. jeotgali* (NZ_AMQY00000000), *H. smyrnensis* (NZ_AJKS00000000), *H. stevensii* (NZ_AJTS00000000), *H. titanicae* (NZ_AOPO00000000), *H. zhanjiangensis* (NZ_ARIT00000000).

As a halophilic bacterium, the genome of *H. lutea* also shows properties related to solute and ion transport, 203 genes related ion transport and metablism, 60 genes related TRAP-type C4-dicarboxylate transport system which is a crucial family of solute transporters. Moreover, nitrate reduction was tested using API 20NE system and 57 genes were predicted to participate in the nitrogen metabolism. PTS IIA-like nitrogen-regulatory protein, nitrate and sulfonate transport systems related genes were also detected in its genome.

## Conclusions

The genome sequence and annotation of *H. lutea* YIM 91125^T^ were presented. The genome comprises 42 scaffolds which together represent the organism of approximately 4.53 Mb. It encodes for key genes and pathways involved in the compatible solutes production and nitrogen degradation. This provides clues to discover novel genes and functions, and leads to an improved understanding of halophilic microbial evolution and function in the extremely salty conditions. YIM 91125^T^ participates in nitrogen cycling, although the process of reducing nitrogen needs further studies to fully understand the related pathways. The genome sequencing of *H. lutea* marks an important step toward a comprehensive genomic catalog and the metabolic diversity of halophilic bacteria. It may contribute to further studies on important process for *Halomonas*, such as quorum-sensing regulatory and osmoadaption. Combining with genomes of other members in *Halomonas*, will make an important advance in understanding of the ecological roles that *Halomonas* species play in those hypersaline environments and their relationships with other halophilic and nonhalophilic microorganisms.

## Abbreviations

DDH: DNA-DNA hybridization; HSP: High-scoring segment pair.

## Competing interests

The authors declare that they have no competing interests.

## Authors’ contributions

WJL and HPK conducted the study. XYG performed the data analyses, genome comparison, and wrote the manuscript. XYZ, HWL, YZ, AL, HPK,NCK and WJL participated in writing the manuscript. JH, MH, EL, MH, AP, NNI, KM, BJT, VM and TW performed genome sequencing, assembly and annotation. All authors read and approved the final manuscript.

## Supplementary Material

Additional file 1: Figure S1 Phylogenetic tree of the genus *Halomonas*. Click here for file
