# Supplementary figures and images for: Draft genome sequence of Halomonas lutea strain YIM 91125T (DSM 23508T) isolated from the alkaline Lake Ebinur in Northwest China
Source: Stand Genomic Sci. 2015 Jan 20;10:1. doi: 10.1186/1944-3277-10-1 (PMC4315136; doi:10.1186/1944-3277-10-1)

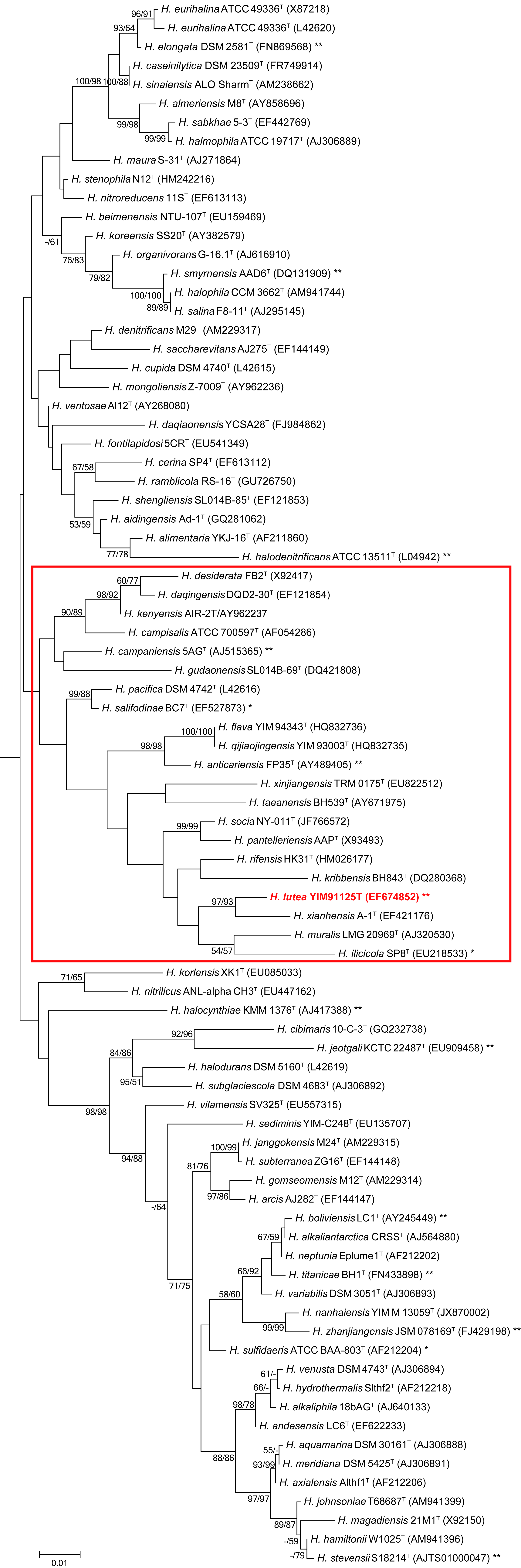

Supplement: Additional file 1: Figure S1 — Phylogenetic tree of the genus Halomonas. [file 1944-3277-10-1-S1.tiff]
